# The long‐term outcomes of maxillary implant‐supported overdentures: A retrospective study

**DOI:** 10.1111/jopr.70093

**Published:** 2026-01-09

**Authors:** Majid Zakeri, Amir Azarpazhooh, Vanessa C. Mendes, Ahmed Ben Suleiman, David Chvartszaid

**Affiliations:** ^1^ Faculty of Dentistry, Department of Prosthodontics University of Toronto Toronto Ontario Canada; ^2^ Department of Dentistry Mount Sinai Hospital Toronto Ontario Canada; ^3^ Faculty of Dentistry, Dental Public Health and Endodontics University of Toronto Toronto Ontario Canada; ^4^ Divisions of Endodontics and Research, Department of Dentistry Mount Sinai Hospital Toronto Ontario Canada; ^5^ Faculty of Dentistry Graduate Periodontics Program University of Toronto Toronto Ontario Canada; ^6^ Graduate Prosthodontics Program University of British Columbia Vancouver British Columbia Canada; ^7^ Alpha Omega Dental Center Baycrest Health Sciences Toronto Ontario Canada

**Keywords:** biologic complication, dental implant, overdenture, prosthodontic maintenance, technical complication

## Abstract

**Purpose:**

This study examined the survival rate of implants and maxillary implant‐supported overdentures (MIS‐ODs) and identified the frequency of maintenance needs as well as technical and biological complications associated with MIS‐ODs.

**Materials and Methods:**

This retrospective cohort study included 86 patients who received 86 MIS‐ODs between 1982 and 2023 in a university setting. Data analyses were performed to determine the survival rate of implants and MIS‐ODs, as well as the frequency of maintenance events and complications (technical and biological). The survival proportion of MIS‐ODs was estimated, and prognostic factors were analyzed using Cox regression analyses (*p* < 0.05).

**Results:**

The median follow‐up time for MIS‐ODs in this study was 39.0 months (interquartile range [IQR]: 15.0–84.3). Among 86 MIS‐ODs, 45.3% had non‐splinted attachments and 54.7% had splinted attachments; 9.3% of prostheses were supported by 2 to 3 implants, 59.3% by 4 implants, and 31.4% by more than 4 implants. The most common maintenance intervention was adjustment of the prosthesis intaglio surface. Technical complications occurred in nearly 60% of cases, with inadequate prosthesis retention being the most frequently reported issue. The most prevalent biological complication was peri‐implant mucositis. Lastly, 27.9% of prostheses (24 cases) required a new prosthesis after a median survival time of 58.5 months (IQR: 16.0–79.0) due to acrylic tooth wear, implant loss, fabrication errors, and suboptimal retention.

**Conclusions:**

Within the limitations of this study, MIS‐OD survival is independent of the prosthesis design characteristics. However, certain technical and biological complications can affect the survival of MIS‐ODs.

Despite recent advances in dental care, the global prevalence of edentulism is expected to remain stable at approximately 250 million individuals by 2050, primarily due to population aging, particularly among those aged 65 years and older.^1^ Edentulism has been associated with substantial impacts on oral health‐related quality of life, altered dietary and nutritional intake, and overall well‐being.^2^, ^3^ Furthermore, it frequently coexists with systemic comorbidities that may further compromise general health.^2^, ^4^

Several treatment modalities are available for the rehabilitation of the edentulous maxilla. In most cases, a maxillary removable complete denture provides acceptable function and patient satisfaction.^1^ However, such prostheses are often associated with limitations, including difficulties with phonetics, compromised esthetics, gagging, inadequate retention, and frequent maintenance needs.^1^ Patients who remain dissatisfied with removable complete dentures may be considered for implant‐supported prosthetic alternatives, such as implant‐supported overdentures (IS‐ODs) or implant‐supported fixed dental prostheses (IS‐FDPs), which offer improved function and patient‐centered outcomes.^2^, ^3^

Maxillary IS‐ODs (MIS‐ODs) offer several advantages over conventional removable complete dentures, including reduced palatal coverage, enhanced retention and stability, and improved chewing function.^2^, ^3^ Compared to maxillary IS‐FDPs (MIS‐FDPs), MIS‐ODs additionally provide lip support, improved speech comfort, hygiene accessibility, lower cost, and reduced impact from future implant loss.^5^, ^6^, ^7^ However, like all prosthodontic treatments, MIS‐ODs require ongoing maintenance and are susceptible to technical complications involving wear or fracture of prosthetic components, as well as biologic complications including peri‐implant mucositis, peri‐implantitis, and implant failure.^8^, ^9^, ^10^ Payne et al. defined routine maintenance as the typical procedure that includes adjustment or replacement of attachment retention elements or contour adjustments to preserve proper function.^11^ The frequency of MIS‐OD maintenance and the occurrence of complications may vary according to prosthesis design, attachment type, the number and distribution of supporting implants, and adherence to postdelivery maintenance protocols.^8^

Most studies pertaining to MIS‐ODs focus primarily on implant treatment outcomes and biologic complications.^12^ There is limited consensus in the literature regarding the frequency and etiology of technical and biologic complications associated with MIS‐ODs, with significant variability in study methodologies, including follow‐up duration and prosthesis‐ and implant‐related factors.^13^ Although several studies have reported prosthetic complications associated with MIS‐ODs,^14^ definitive conclusions regarding their long‐term outcomes remain difficult to establish.

This study aimed to evaluate the survival of MIS‐ODs and their supporting implants and to determine the frequency of maintenance needs, as well as the occurrence of technical and biologic complications associated with MIS‐ODs. We hypothesized that the survival of MIS‐OD prostheses and their supporting implants is not significantly associated with prosthesis design characteristics or the occurrence of technical and biologic complications.

## MATERIALS AND METHODS

This single‐center retrospective cohort study reviewed dental records of patients who received MIS‐ODs in a university setting between January 1982 and October 2023. The study was approved by the Research Ethics Board (protocol No. 44011).

The inclusion criteria for this study required patients with an edentulous maxilla who had been rehabilitated with MIS‐ODs and had at least one follow‐up appointment after a minimum of 1 year of functional loading.^15^ Patients were excluded if they did not complete a 1‐year follow‐up after receiving MIS‐ODs. However, cases that failed within the initial 12 months were still included in the analysis. No patients were excluded based on medical condition.^15^

A systematic search was conducted to identify eligible patient records using keywords corresponding to treatment codes related to MIS‐ODs within the electronic health record database (axiUm; Exan Enterprises Inc., Surrey, BC, Canada). Patient daily clinical notes, intraoral photographs, radiographs, and laboratory scripts were meticulously reviewed to confirm the presence of MIS‐ODs. Verified cases were then systematically documented using a structured electronic data collection form (Google Sheets; Google LLC, USA). Data were collected on patient demographics, follow‐up nature, opposing dentition, type of attachment mechanism, implant characteristics, prosthesis details, prosthodontic maintenance, and the occurrence of technical and biologic complications following prosthesis insertion (Table 1).

**TABLE 1:** The univariate distribution of investigated variables in the study population (*n* = 86).

Variable	Level	Frequency	Percentage
**A. Patient‐level factors**			
Age	< 65 years	35	40.7%
	≥ 65 years	51	59.3%
Gender	Male	43	50.0%
	Female	43	50.0%
ASA	I	48	55.8%
	II	38	44.2%
Smoking	Yes	22	25.6%
Osteoporosis	Yes	8	9.3%
Diabetes	Yes	9	10.5%
Follow‐up nature	Regular follow‐up	22	25.6%
	Irregular follow‐up^a^	64	74.4%
Follow‐up time	Median (*m*) (IQR; mean ± SD)	39.0 (15.0–84.3; 64.8 ± 78.2)	
**B. Prosthesis‐level factors**			
Opposing dentition	Natural dentition/fixed prosthesis(es)	33	38.4%
	Natural dentition/fixed prosthesis(es) + RPD	27	31.4%
	IS‐OD	26	30.2%
Number of implants supporting MIS‐OD	2 to 3 implants	8	9.3%
	4 implants	51	59.3%
	> 4 Implants	27	31.4%
Initial prosthesis plan	Planned for MIS‐OD	72	83.7%
	Planned for IS‐FDP^b^	14	16.3%
Type of attachment mechanism	Non‐splinted attachment	39	45.3%
	Splinted attachment mechanism (i.e., bar)	47	54.7%
Bar framework material (*n* = 47)^c^	Titanium	30	63.8%
	Chrome‐Cobalt	5	10.6%
	Gold IV	12	25.5%
Bar fabrication method (*n* = 47)^c^	Milled	30	63.8%
	Cast	17	36.2%
Presence of distal cantilever (*n* = 47)^c^	Yes	25	53.2%
Palatal coverage	Yes	25	29.1%
Denture base reinforcement	Yes	72	83.7%
**C. Maintenance events**			
Number of maintenance events	0	23	26.7%
	1	40	46.6%
	> 1	23	26.7%
Time to the first maintenance event (month)	Median (*m*) (IQR; mean ± SD)	12.0 (6.0–25.0; 26.3 ± 48.2)	
Adjustment of retention mechanism	Yes	42	48.8%
Replacement of worn components of retention mechanism	Yes	9	10.5%
Adjustment of intaglio surface of prosthesis	Yes	41	47.7%
**D. Technical complications**			
Number of technical complication events	0	35	40.7%
	1	18	20.9%
	> 1	33	38.4%
Time to the first technical complication (month)	Median (*m*) (IQR; mean ± SD)	17.0 (5.5–38.0; 34.9 ± 60.9)	
**E. Types of technical complications**			
Fracture of denture base	Yes	12	14.0%
Fracture of acrylic tooth	Yes	20	23.3%
Compromised function as a result of suboptimal retention	Yes	31	36.0%
Food impaction inside the housing/abutment preventing prosthesis seating	Yes	3	3.5%
Excessive retention	Yes	3	3.5%
Acrylic tooth wear	No wear	62	72.1%
	Mild	9	10.5%
	Moderate	9	10.5%
	Severe	6	7%
Loosening of prosthetic screw	Yes	1	1.2%
Loosening of abutment screw	Yes	3	3.5%
Fracture of abutment screw	No	86	100%
Loosening of abutment	Yes	2	2.3%
Dislodgment/loosening of abutment housing	Yes	1	1.2%
Damage (iatrogenic) to locator housing	Yes	3	3.5%
Fracture of ball abutment on top of bar	Yes	1	1.2%
Fracture of clip	Yes	4	4.7%
Fracture of framework	Yes	1	1.2%
Phonetic deficiency	Yes	3	3.5%
Compromised esthetics	Yes	6	7.0%
**F. Management of technical complications**			
Repair of fracture	Simple repair of acrylic tooth and/or denture base	21	24.4%
	Repair with metal reinforcement	3	3.5%
Reline (processed reline) of prosthesis	Yes	16	18.6%
Refurbishment of prosthesis	Yes	4	4.7%
Replacement of abutment	Yes	2	2.3%
Replacement of bar/framework	Yes, using the same design and material	1	1.2%
	Yes, using different designs and materials	2	2.3%
Change of the attachment mechanism	Yes	1	1.2%
Addition of palatal coverage	Yes	3	3.5%
Addition of implant to increase retention	Yes	2	2.3%
Re‐pick up existing housing/retentive housing/clip/gold cap	Yes	14	16.3%
Prosthesis survival time	Median (*m*) (IQR; mean ± SD)	39.0 (15.0–84.3; 64.8 ± 78.2)	
**G. Biological complications**			
Number of biological complication events	0	38	44.2%
	1	17	19.8%
	> 1	31	36.0%
Time to the first biological complication (months)	Median (*m*) (IQR; mean ± SD)	24.5 (8.0–53.3; 50.9 ± 72.6)	
**H. Diagnosis of biological complications**			
Peri‐implant mucositis	Yes	47	54.7%
Peri‐implantitis	Yes	36	41.9%
Marginal bone loss (relative to implant‐abutment junction, mm)	1–2 mm	8	9.3%
	3–4 mm	14	16.3%
	> 4 mm	21	24.4%
Implant failure before loading the prosthesis	Yes	16	18.6%
Implant failure after loading the prosthesis	Yes	12	14.0%
**I. Management of biological complications**			
Non‐surgical therapy	Yes	38	44.2%
Surgical therapy (excluding implant removal and implant placement)	Yes	6	7.0%
Implant removal	Yes	21	24.4%
Implant placement	Yes	7	8.1%
Implant survival time	Median (*m*) (IQR; mean ± SD)	67.0 (27.3–122.0; 87.2 ± 81.9)	

Abbreviations: ASA, American Society of Anesthesiologists; IQR, interquartile rate; SD, standard deviation.

^a^ Seen only for an occasional follow‐up or for emergency.

^b^ Planned for IS‐FDP, but MIS‐OD was ultimately provided.

^c^ *n* = 47 indicated the number of prostheses with splinted attachment.

### Statistical analysis

To estimate the required sample size, we applied the general rule of five events per predictor variable to minimize the risk of overfitting.^16^, ^17^ Based on a systematic review reporting prosthesis survival rates for MIS‐ODs ranging from 75% to 100%,^18^ we conservatively assumed a 15% failure rate and expected five potential predictors.^8^ The model would therefore require 25 events (5 predictors × 5 events per variable), corresponding to a minimum sample size of 166 prostheses (25 ÷ 0.15). This approach balances statistical rigor with feasibility in the clinical setting.

The data collected through the structured electronic form were imported into IBM SPSS Statistics (Version 27 for Mac; IBM Corp, Chicago, IL) for analysis. Descriptive statistics were computed, including means, standard deviations, medians, and interquartile ranges (IQRs) for continuous variables, and frequencies for dichotomous variables. No imputation was performed to address missing data. The frequency of post‐insertion maintenance events and complications, categorized as either technical or biologic, was calculated and reported as the rate of maintenance and complications per prosthesis. Each patient in the study contributed a single MIS‐OD; therefore, no within‐patient clustering was present. For survival analyses, Kaplan–Meier survival curves and unadjusted Cox regression analyses were performed to evaluate differences in prosthesis survival time. Survival rates were reported at 5, 10, and 15 years based on follow‐up distribution, with the number at risk included for transparency. Results were expressed as hazard ratios (HRs) with corresponding 95% confidence intervals (CIs) and log‐rank (Mantel–Cox) *p*‐values. Multivariable Cox proportional hazards regression was conducted to identify significant predictors of prosthesis failure. Variables were retained in the final model if they achieved statistical significance (*p* ≤ 0.05) or demonstrated a substantial effect size (HR or OR > 1.5 or < 0.5) with *p*‐values ≤ 0.10. The proportional hazards assumption for the Cox model was evaluated using scaled Schoenfeld residuals. All statistical tests were two‐tailed with a significance threshold set at 5%.

## RESULTS

### Descriptive results

**FIGURE 1:**
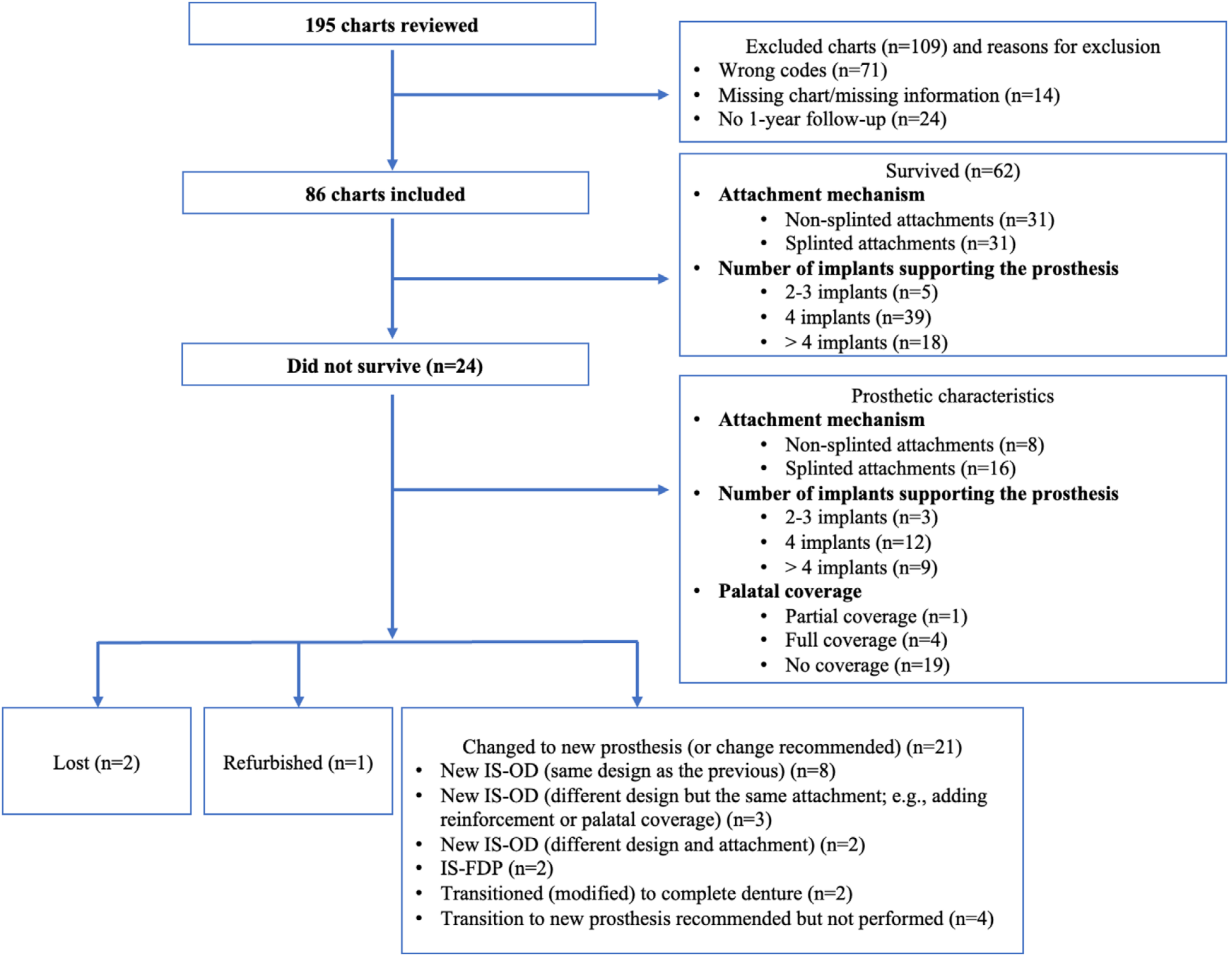
Study flow diagram showing cases that met inclusion criteria and received MIS‐OD.

A total of 195 potential cases of maxillary MIS‐ODs were initially identified. After exclusion of 109 cases not meeting the inclusion criteria (Figure 1), 86 MIS‐ODs were included in the final analysis. Patient and prosthesis characteristics are summarized in Table 1.

#### Patient characteristics

Among the included cohort, 59.3% of patients were aged 65 years or older, with an equal distribution between genders. Most patients were medically classified as ASA I (55.8%) or ASA II (44.2%), indicating generally good systemic health. The majority reported no history of smoking (74.4%), osteoporosis (90.7%), or diabetes (89.5%). However, 74.4% exhibited irregular follow‐up patterns, presenting only for emergencies or sporadic maintenance visits.

#### Prosthesis factors

Opposing dentition most frequently consisted of natural or fixed restorations on natural teeth (38.4%), followed by mixed fixed–removable prostheses (31.4%) and IS‐ODs (30.2%). The majority of MIS‐ODs were supported by four or more implants (90.7%) and were treatment‐planned as such from the outset (83.7%). Splinted attachment systems were used in 54.7% of cases, 70.9% had no palatal coverage, and denture base reinforcement was incorporated in 83.7% of prostheses.

#### Maintenance events

The median time to the first maintenance event was 12.0 months (IQR: 6.0–25.0; mean ± SD: 26.3 ± 48.2 months). Approximately one quarter of prostheses (26.7%) required no maintenance, 46.6% required a single visit, and 26.7% required multiple appointments. The most common maintenance procedures involved adjustments to the intaglio surface (47.7%) and the retentive mechanism (48.8%), particularly replacement of worn or lost inserts. Metallic component replacement, such as Locator housings or ball matrices, occurred in 10.5% of cases (Table 1C).

#### Technical complications and management

The median time to the first technical complication was 17.0 months (IQR: 5.5–38.0; mean ± SD: 34.9 ± 60.9 months). Of the 86 prostheses, 40.7% experienced no technical complications, whereas 20.9% experienced a single event and 38.4% exhibited multiple complications. The most common complication was compromised prosthesis retention (36.0%), followed by acrylic tooth wear (28.0%), acrylic tooth fracture (23.3%), and denture base fracture (14.0%). Less frequent complications included esthetic dissatisfaction (7.0%), clip failure (4.7%), housing failure (3.5%), abutment loosening (2.3%), screw loosening (1.2%), and bar component fracture (1.2%) (Table 1D,E).

Management was tailored to the type of complication. Denture base and acrylic tooth fractures were repaired in 24.4% of cases, with base reinforcement added in 3.5%. Loss of retention was managed through relining (18.6%), implant addition (2.3%), incorporation of palatal coverage (3.5%), or modification of attachment design (1.2%). Acrylic tooth wear was treated by refurbishment (4.7%). Other corrective measures included abutment replacement (2.3%), bar or framework replacement (3.5%), and re‐pickup of retentive components such as housings, clips, or gold caps (16.5%).

#### Biologic complications and management

The median time to the first biologic complication was 24.5 months (IQR: 8.0–53.3; mean ± SD: 50.9 ± 72.6). Among the 86 MIS‐ODs, 44.2% did not experience any biologic complications, while 19.8% experienced one, and 36.0% experienced multiple. The most frequent biologic complications were peri‐implant mucositis (54.7%) and peri‐implantitis (41.9%). Radiographic bone loss of 1–2 mm, 3–4 mm, and > 4 mm was observed in 9.3%, 16.3%, and 24.4% of cases, respectively. Implant failure prior to loading occurred in 18.6% of cases, and failure after loading occurred in 14.0% (Table 1G,H).

Most biologic complications were managed non‐surgically (44.2%), whereas 7.0% required surgical intervention involving implant removal and (re)placement. In 24.4% of cases, one or more implants were removed, either before or after prosthesis loading. Following a median follow‐up of 67.0 months (IQR: 27.3–122.0; mean ± SD: 87.2 ± 81.9 months) (Table 1I), the implant survival rate at the prosthesis level was 86.0%.

#### Transition to new prosthesis

At follow‐up, 62 prostheses (72.1%) remained functional, with a median follow‐up duration of 39.0 months (IQR: 15.0–84.3; mean ± SD: 64.8 ± 78.2). Twenty‐four prostheses (27.9%) failed or were deemed clinically inadequate and were either replaced or recommended for replacement. Among these, one underwent refurbishment, two were lost, 17 were transitioned to new prostheses, and four were recommended for transition but not pursued.

Of the transitioned cases, 87.5% involved fabrication of a completely new prosthesis, whereas 4.1% were modified existing MIS‐ODs with processed relines and added palatal coverage due to complete implant loss. Among 17 newly fabricated prostheses, eight retained the same design and attachment, three maintained the attachment but altered the design, and two changed both. Two cases were converted to implant‐supported fixed dental prostheses (IS‐FDPs), and two were transitioned to conventional complete dentures.

### Survival analysis for prostheses

Survival analysis was performed at the prosthesis level, as biologic complications and implant survival data were recorded per prosthesis. Among 86 MIS‐ODs, 24 (27.9%) were classified as failures (either transitioned or recommended for transition), yielding an overall prosthesis survival rate of 72.1%.

The median follow‐up time was 39.0 months (IQR: 15.0‐84.3; mean ± SD: 64.8 ± 78.2). The median time to failure was 58.5 months (IQR: 16.0–79.0; mean ± SD: 69.85 ± 89.32). Reported reasons for failure included occlusal wear (*n* = 6), implant loss (*n* = 5), fabrication error (*n* = 4), suboptimal retention (*n* = 3), prosthesis loss (*n* = 2), significant adaptation challenges (*n* = 2), denture base fracture (*n* = 1), and unclear justification or missing information (*n* = 1) (Table 2).

**TABLE 2:** Summary of MIS‐ODs that were transitioned or recommended for transition to a new prosthesis among the 86 MIS‐ODs included in the study.

Reason for transition to new prosthesis	Number of prostheses affected out of 86 (%)	Transition to a new prosthesis performed	Transition to new prosthesis recommended but NOT performed
Implant loss	5 (5.8%)	4	1
Significant adaptation challenge	2 (2.3%)	1	1
Denture base fracture/history of fracture/catastrophic fracture with missing segments	1 (1.2%)	1	0
Suboptimal retention	3 (3.5%)	2	1
Occlusal wear	6 (6.9%)	5	1
Prosthesis loss	2 (2.3%)	2	0
Fabrication error	4 (4.7%)	4	0
No clear justification for remake/missing information	1 (1.2%)	1	0
Total	*N*: 24 (27.9%)	20 (23.2%)	4 (4.7%)

Unadjusted Cox regression analyses revealed several variables significantly associated with prosthesis survival (*p* < 0.05): acrylic tooth wear, compromised esthetics, peri‐implant mucositis, implant failure before loading, implant failure after loading, and management approaches such as implant replacement and non‐surgical therapy (Table 3).

**TABLE 3:** The unadjusted effects of study variables on the survival of MIS‐ODs using Cox regression analysis.

Variables	Level	Total *N* (% survived)	Median survival (95% CI)	*p*‐Value (log‐rank test)	HR (95% CI)	*p*‐Value (Wald test)
**Patient‐level factors**						
Age	< 65 years	35 (74.3%)	28.0 (6.9–49.1)	0.370	1	0.376
	≥ 65 years	51 (64.7%)	64.0 (32.0–96.0)		0.6 (0.2–1.5)	
Gender	Male	43 (69.8%)	61.0 (39.1–82.9)	0.577	1	0.580
	Female	43 (67.4%	37.0 (12.4–61.6)		1.2 (0.5–2.6)	
ASA	I	48 (72.9%)	57.0 (27.9–86.1)	0.138	1	0.146
	II	38 (63.2%)	66.5 (39.8–93.1)^a^		1.7 (0.8–3.8)	
Smoking	No	64 (70.3%)	57.0 (33.2–80.8)	0.155	1	0.164
	Yes	22 (63.6%)	38.0 (11.8–64.2)		1.8 (0.7–4.3)	
Osteoporosis	No	78 (79.5%)	45.0 (24.7–65.3)	0.860	1	0.861
	Yes	8 (50.0%)	95.8 (16.8–174.7)^a^		1.1 (0.3–3.2)	
Diabetes	No	77 (68.8%)	45.0 (25.6–64.4)	0.256	1	0.269
	Yes	9 (66.7%)	57.0 (21.2–92.8)		2.0 (0.5–6.9)	
Follow‐up nature	Regular follow‐up	22 (72.7%)	69.0 (36.0–102.0)	0.413	1	0.418
	Irregular follow‐up	64 (67.2%)	38.0 (3.2–72.8)		1.4 (0.5–3.6)	
**Prosthesis‐level factors**						
Opposing dentition	Natural/fixed + RPD	33 (57.6%)	57.0 (18.8–95.2)	0.140	1	
	Natural/fixed + RPD	27 (66.7%)	66.0 (7.3–124.7)		0.5 (0.2–1.1)	0.120
	IS‐OD	26 (84.6%)	49.5 (34.5–64.5)^a^		0.4 (0.1–1.2)	0.126
Number of implants supporting MIS‐OD	2–3 implants	8 (62.5%)	360.0 (0.0–738.0)^a^	0.200	1	
	4 implants	51 (74.5%)	35.0 (13.5–56.5)		6.5 (0.7–60.2)	0.099
	> 4 implants	27 (59.3%)	108.0 (23.9–192.1)		5.6 (0.6–48.7)	0.114
Initial prosthesis plan	Planned for MIS‐OD	72 (73.6%)	38.0 (20.5–55.5)	0.189	1	0.197
	Planned for MIS‐FDP (but MIS‐OD ultimately provided)	14 (42.9%)	151.0 (58.2–243.8)		1.7 (0.7–4.1)	
Type of attachment mechanism	Non‐splinted	39 (71.8%)	41.0 (30.2–51.8)	0.420	1	0.424
	Splinted attachment (i.e., bar)	47 (66.0%)	57.0 (24.3–89.7)		0.7 (0.3–1.5)	
Bar framework material (*n* = 47)	Titanium	30 (73.3%)	177.0 (48.9–305.1)	0.740	1	
	CoCr	5 (20.0%)	150.0 (–)		1.5 (0.4–5.6)	0.489
	Gold IV	12 (66.7%)	186.0 (–)		0.9 (0.2–3.4)	0.966
Bar fabrication method (*n* = 47)	Milled	30 (73.3%)	177.0 (48.9–305.1)	0.730	1	0.735
	Cast	17 (52.9%)	186.0 (91.6–280.4)		1.1 (0.4–3.3)	
Presence of distal cantilever (*n* = 47)	No	22 (72.7%)	177.0 (122.7–231.3)	0.120	1	0.131
	Yes	25 (60.0%)	92.0 (59.3–124.7)		2.3 (0.7–6.7)	
Palatal coverage	No	61 (67.2%)	41.0 (13.8–68.2)	0.130	1	0.142
	Yes	25 (72.0%)	61.0 (30.6–91.4)		0.5 (0.1–1.2)	
Denture base reinforcement	No	14 (57.1%)	61.0 (51.5–70.5)	0.470	1	0.474
	Yes	72 (70.8%)	35.0 (12.2–57.8)		1.4 (0.5–3.9)	
**Maintenance**						
Maintenance event	No	23 (78.3%)	89.0 (0.0–393.6)	0.660	1	0.663
	Yes	63 (65.1%)	151.0 (72.8–229.2)		0.8 (0.2–2.1)	
Adjustment of intaglio surface of prosthesis	No	45 (75.6%)	20.0 (7.4–32.6)	0.440	1	0.442
	Yes	41 (61.0%)	61.0 (26.3–95.7)		1.3 (0.6–3.1)	
Adjustment of retention mechanism	No	44 (68.2%)	90.0 (68.8–111.2)	0.110	1	0.114
	Yes	42 (69.0%)	186.0 (119.2–252.8)		0.5 (0.2–1.1)	
Replacement of worn components of retention mechanism	No	77 (66.2%)	150.0 (51.3–248.7)	0.080	1	0.118
	Yes	9 (88.9%)	–		0.2 (0.0–1.5)	
**Technical complication**						
Technical complication	No	35 (88.6%)	17.0 (12.9–21.1)	0.880	1	0.879
	Yes	51 (54.9%)	86.0 (61.0–111.0)		0.9 (0.2–2.8)	
**Type of technical complication**						
Fracture of denture base	No	74 (74.3%)	187.0 (26.3–347.7)	0.490	1	0.496
	Yes	12 (33.3%)	94.0 (42.1–145.9)		1.3 (0.5–3.1)	
Fracture of acrylic tooth	No	66 (72.7%)	28.0 (7.0–49.0)	0.490	1	0.490
	Yes	20 (55.0%)	70.0 (39.8–100.2)		1.3 (0.5–3.0)	
Inadequate retention	No	55 (78.2%)	20.0 (7.2–32.8)	0.360	1	0.366
	Yes	31 (51.6%)	86.0 (52.3–119.7)		1.4 (0.6–3.1)	
Excessive retention	No	83 (68.7%)	45.0 (25.7–64.3)	0.910	1	0.907
	Yes	3 (66.7%)	63.0 (5.0–121.0)^a^		1.1 (0.1–8.3)	
Acrylic tooth wear	No wear	62 (74.2%)	22.0 (11.7–32.3)	**0.010** ^b^	1	**0.015** ^b^
	Yes	24 (54.2%	117.0 (94.4–139.6)		0.2 (0.1–0.7)	
Loosening of prosthetic screw	No	85 (69.4%)	44.0 (23.7–64.3)	**0.020** ^b^	1	0.053
	Yes	1 (0.0%)	109.0 (46.6–171.4)		7.7 (0.9–62.4)	
Damage (iatrogenic) to locator housing	No	83 (68.7%)	45.0 (23.6–66.4)	0.960	1	0.963
	Yes	3 (66.7%)	58.0 (35.6–80.4)		0.9 (0.1–7.0)	
Fracture of ball abutment on top of bar	No	85 (68.2%)	57.0 (36.3–77.7)	0.700	1	0.800
	Yes	1 (100.0%)	35.0^a^		0.04 (0–694,686,290.3)	
Fracture of clip	No	82 (69.5%)	45.0 (25.8–64.2)	0.870	1	0.866
	Yes	4 (50.0%)	15.0 (0.0–59.1)^a^		1.1 (0.2–5.1)	
Framework fracture	No	85 (69.4%)	45.0 (25.8–64.2)	0.090	1	0.127
	Yes	1 (0.0%)	69.0^a^		4.9 (0.6–38.4)	
Phonetic deficiency	No	83 (68.7%)	57.0 (37.7–76.3)	0.550	1	0.561
	Yes	3 (66.7%)	45.0 (0.0–93.0)^a^		0.5 (0.0–4.2)	
Compromised aesthetics	No	80 (71.3%)	57.0 (33.6–80.4)	**0.010** ^b^	1	**0.017** ^b^
	Yes	6 (33.3%)	38.0 (9.2–66.8)		3.7 (1.2–11.3)	
**Management of technical complications**						
Repair fracture	No	62 (74.2%)	28.0 (9.5–46.5)	0.570	1	0.573
	Yes	24 (54.2%)	70.0 (40.8–99.2)		1.2 (0.5–2.8)	
Reline (processed)	No	70 (71.4%)	37.0 (19.3–54.7)	0.310	1	0.314
	Yes	16 (56.3%)	111.0 (102.5–119.5)		0.6 (0.2–1.5)	
Refurbishment of prosthesis	No	82 (68.3%)	57.0 (35.5–78.5)	0.560	1	0.566
	Yes	4 (75.0%)	40.0 (8.6–71.4)		1.8 (0.2–14.1)	
Replacement of bar/framework	No	83 (68.7%)	45.0 (25.9–64.1)	0.770	1	0.768
	Yes	3 (66.7%)	79.0^a^		1.3 (0.1–10.2)	
Change of the attachment mechanism	No	85 (69.4%)	45.0 (25.8–64.2)	0.060	1	0.098
	Yes	1 (0.0%)	69.0^a^		5.6 (0.7–44.5)	
Addition of palatal coverage	No	83 (68.7%)	57.0 (37.5–76.5)	0.500	1	0.511
	Yes	3 (66.7%)	45.0 (29.0–61.0)		1.9 (0.2–15.4)	
Addition of implant to increase retention	No	84 (69.0%)	45.0 (27.3–62.7)	0.440	1	0.451
	Yes	2 (50.0%)	16.0^a^		2.1 (0.2–16.4)	
Re‐pick up existing housing/retentive housing/clip/gold cap	No	72 (66.7%)	41.0 (15.7–66.3)	0.290	1	0.306
	Yes	14 (78.6%)	45.0 (21.4–68.6)		0.5 (0.1–1.7)	
**Biological complication**						
Biological complications	0	38 (73.7%)	66.0 (38.6–93.4)	0.070	1	0.074
	1	48 (64.6%)	151.0 (51.5–250.5)		0.4 (0.2–1.0)	
**Diagnosis of biological complication**						
Peri‐implant mucositis	No	39 (71.8%)	86.0 (66.0–106.0)	**0.040** ^b^	1	**0.049** ^b^
	Yes	47 (66.0%)	179.0 (71.1–286.9)		0.4 (0.1–0.9)	
Peri‐implantitis	No	50 (72.0%)	86.0 (64.6–107.4)	0.120	1	0.127
	Yes	36 (63.9%)	179.0 (64.1–293.9)		0.5 (0.2–1.1)	
Marginal bone loss (relative to implant‐abutment junction, mm)	0–2 mm	51 (76.5%)	94.0 (49.3–138.7)	0.490	1	0.495
	> 2mm	35 (57.1%)	151.0 (41.3–260.7)		0.7 (0.3–1.6)	
Implant failure before loading the prosthesis	No	70 (74.3%)	179.0 (138.0–220.0)	**< 0.001** ^b^	1	**0.002** ^b^
	Yes	16 (43.8%)	65.0 (37.6–92.4)		3.8 (1.6–9.0)	
Implant failure after loading the prosthesis	No	74 (74.3%)	179.0 (68.0–290.0)	**0.040** ^b^	1	**0.049** ^b^
	Yes	12 (33.3%)	51.0 (25.5–76.5)		2.3 (1.0–5.5)	
**Management of biological complications**						
Nonsurgical therapy	No	48 (68.8%)	65.0 (56.4–73.6)	**< 0.001** ^b^	1	**0.001** ^b^
	Yes	38 (68.4%)	179.0^a^		0.2 (0.1–0.5)	
Surgical therapy (excluding implant removal and implant placement)	No	80 (70.0%)	151.0 (65.9–236.1)	0.760	1	0.757
	Yes	6 (50.0%)	22.0^a^		0.8 (0.2–2.8)	
Implant removal	No	65 (73.8%)	179.0 (112.3–245.7)	0.050	1	0.055
	Yes	21 (52.4%)	65.0 (30.1–99.9)		2.1 (0.9–4.8)	
Implant placement	No	79 (70.9%)	151.0 (43.0–259.0)	**< 0.001** ^b^	1	**< 0.001** ^b^
	Yes	7 (42.9%)	22.0 (17.7–26.3)		8.0 (2.3–27.6)	

*Note*: Median survival times are reported with 95% confidence intervals derived from Kaplan–Meier estimates, not from raw data distributions.

Abbreviations: ASA, American Society of Anesthesiologists; CI, confidence interval; HR, hazard ratio.

^a^ Median not reached; mean reported instead.

^b^ Statistically significant (*p* < 0.05) values are shown in bold.

– Median not available due to insufficient events; mean, 95% CI, and log‐rank test not interpretable from a single censored case.

**FIGURE 2:**
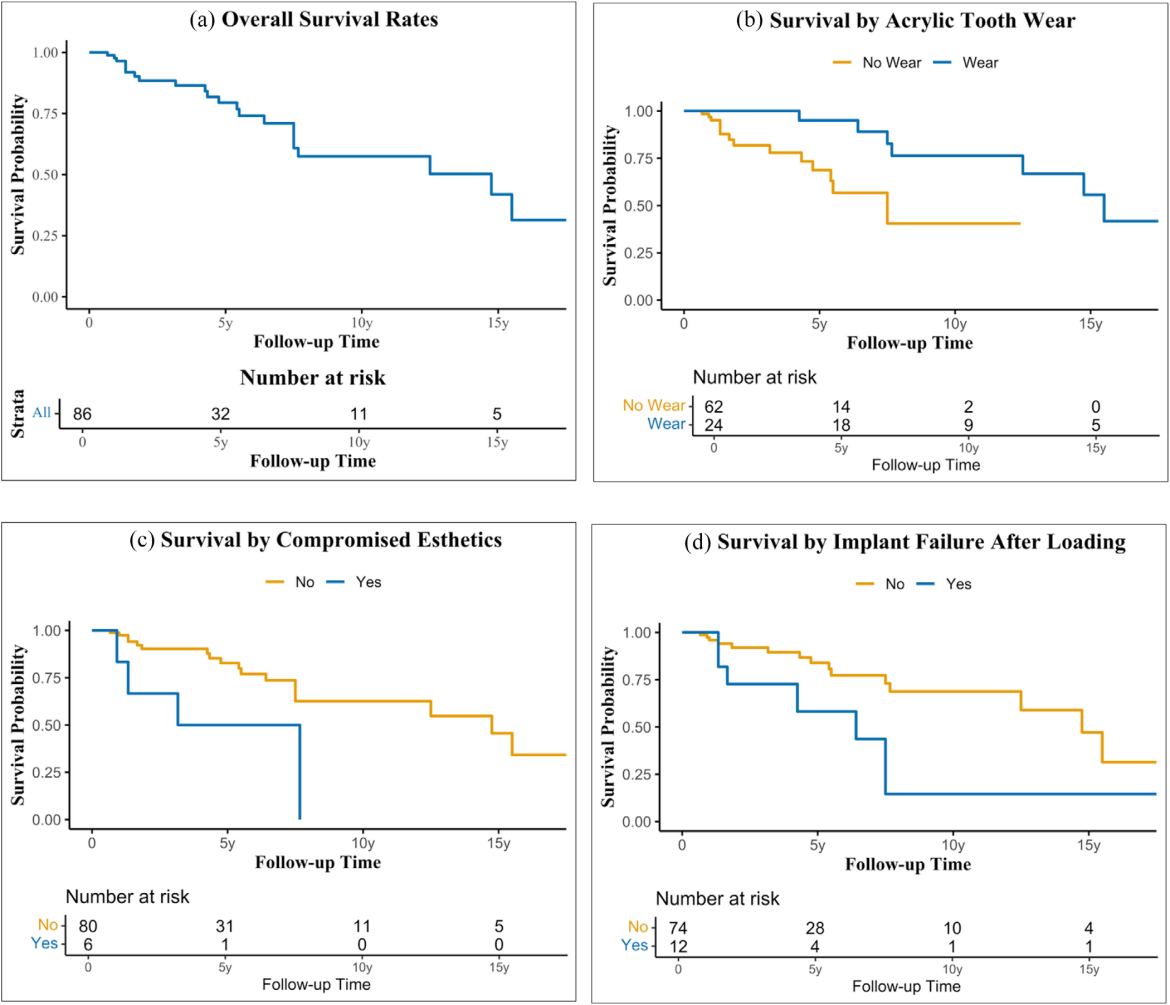
Kaplan–Meier survival curves for overall survival time and predictors of MIS‐ODs. Note: the figure shows the overall survival rates (a) and Kaplan–Meier curves according to the 3 significant predictors of percentage acrylic tooth wear (b), compromised esthetics (c), and implant failure after loading the prostheses (d).

For multivariable modeling, variables suggesting reverse causality or conceptual overlap with outcome events (e.g., non‐surgical therapy, implant replacement) were excluded. Implant failure before loading the prosthesis and peri‐implant mucositis were also excluded since they may be influenced by factors independent of prosthesis design, including poor oral hygiene for peri‐implant mucositis^12^ and a higher rate of implant failure in the maxilla due to decreased density and quantity of bone.^13^ The final multivariable analysis (Table 4, Figure 2) identified compromised esthetics and implant failure after loading as unfavorable predictors, while acrylic tooth wear emerged as a favorable predictor of prosthesis survival.

**TABLE 4:** The results of the adjusted multivariate regression analysis: multivariate Cox regression predicting the outcome of MIS‐OD survival.

Prognostic factors	Level	HR (95% CI)	*p*‐Value^*^
Acrylic tooth wear (Ref: No)	Yes	0.2 (0.0 – 0.6)	**0.004** ^a^
Compromised esthetics (Ref: No)	Yes	6.6 (2.0 – 21.9)	**0.002** ^a^

^a^ Statistically significant (*p* < 0.05).

* *p*‐Value generated by multivariate Cox proportional hazards regression.

Kaplan–Meier analysis demonstrated prosthesis survival rates of 79.5%, 57.5%, and 41.9% at 5, 10, and 15 years, respectively (Figure 2a). Higher survival was associated with acrylic tooth wear and the absence of esthetic compromise, whereas implant failure after loading significantly reduced survival (Figure 2b–d). In the initial multivariable model, the proportional hazards assumption was not met for implant failure (*p* = 0.013; global *p* = 0.045). To address this, implant failure was stratified in the final Cox model, which included acrylic tooth wear and compromised esthetics. The revised model satisfied the proportional hazards assumption (global *p* = 0.28). In the adjusted Cox regression analysis, acrylic tooth wear was significantly associated with improved prosthesis survival (HR = 0.2; 95% CI, 0.0–0.6; *p* = 0.004), whereas compromised esthetics was a strong predictor of failure (HR = 6.6; 95% CI, 2.0–21.9; *p* = 0.002).

## DISCUSSION

This retrospective study evaluated the longevity, maintenance requirements, and complications associated with MIS‐ODs and their supporting implants. A retrospective design was deemed the most suitable method for assessing long‐term outcomes and identifying potential risk factors, as it allows analysis of large cohorts with extended follow‐up periods while minimizing loss to follow‐up bias.^19^ The present findings contribute to the growing body of evidence on MIS‐OD performance, emphasizing prosthesis survival, maintenance frequency, and the influence of technical and biologic complications on clinical outcomes.

The overall prosthesis survival rate of 72.1% observed in this cohort is lower than those reported in previous studies, with survival rates of 79.5% at 5 years, 57.5% at 10 years, and 41.9% at 15 years.^20^, ^21^ Several factors may account for this discrepancy, including the relatively large sample size, extended observation period, and higher incidence of complications. Additionally, the irregular follow‐up pattern observed in 74.4% of patients may have contributed to delayed diagnosis and management of early technical or biologic problems. Despite these challenges, the survival rate of supporting implants remained relatively high (86.0%), suggesting that most failures were prosthetic rather than implant‐related.^21^, ^22^

### Maintenance events

The median time to the first maintenance event was 12 months, a finding consistent with previous studies indicating that maintenance demands peak during the early years of service.^14^, ^22^ Adjustments to the intaglio surface and attachment components were the most common maintenance procedures to improve fit and comfort.^22^, ^23^

Non‐splinted attachment systems required more frequent maintenance, primarily due to wear of retentive components such as locator inserts and ball matrices, while splinted bar designs required fewer maintenance interventions. This observation is consistent with existing literature, which suggests that splinted designs may offer improved mechanical stability and reduced component wear over time.^4^, ^20^

A relatively high incidence of maintenance events was recorded, consistent with previous literature.^20^, ^24^, ^25^, ^26^ This finding likely reflects both the inherent design characteristics of MIS‐ODs that may require frequent adjustment to the attachment components and intaglio surface of the prostheses and the extended follow‐up duration.^22^, ^23^ Continuous evolution of attachment systems also complicates direct comparisons, as earlier generations of components demonstrated different wear characteristics than current designs.^22^ No statistically significant association was found between maintenance frequency and prosthetic design, type of attachment, or implant number and distribution, contrasting with Osman et al., who reported that attachment type directly influenced maintenance needs.^22^

#### Technical complications

Technical complications were common, with a median onset time of 17 months. Approximately 59% of MIS‐ODs experienced at least one event, aligning with previous reports in the literature.^9^ These complications encompassed issues such as retention, wear or fracture of acrylic teeth, and fracture of the denture base. These findings underscore the multifactorial nature of technical issues, which may stem from prosthetic design, material properties, opposing occlusion, and treatment planning philosophy. Historically, MIS‐ODs were often used as salvage restorations following failure of planned implant‐supported fixed prostheses (IS‐FDPs), and early designs lacked design standardization or denture base reinforcement, leading to higher complication rates.^3^, ^27^ However, contemporary MIS‐ODs, characterized by reduced palatal coverage and metal reinforcement, have demonstrated fewer complications.^5^

Inadequate retention remains the most frequent technical concern, observed in 36.0% of cases. The literature has attributed this to a range of contributing factors, including the type of attachment mechanism, ^27^ prosthesis design, and the number of supporting implants, ^27^ implant angulation, ^23^, ^28^ and the characteristics of the opposing dentition.^23^ These variables can affect load distribution, wear of retentive components, and long‐term prosthesis stability.

Acrylic tooth wear and fracture were common issues in this study, reported in 27.9% and 23.3% of prostheses, respectively, likely associated with time in function, ^14^ denture habits, parafunctional activities, and opposing dentition.^8^, ^22^ Denture base fracture occurred in 14.0% of cases, often associated with reduced palatal coverage,^29^, ^30^ limited acrylic thickness, and low fracture strength of the polymethylmethacrylate.^30^, ^31^ Reinforcing the denture base with a metal framework can reduce the risk of denture base fracture.^23^, ^32^

While esthetic outcomes are generally favorable for MIS‐ODs, ^27^ 7% of cases exhibited esthetic compromise related to tooth shade and shape, progressive acrylic tooth wear, and insufficient lip support. These findings reinforce the need for individualized esthetic assessment, particularly in maxillary cases where facial support and appearance significantly affect patient satisfaction.^27^

This study also identified less frequent but clinically relevant technical issues that can arise with MIS‐ODs in the long term, such as prosthetic and abutment screw loosening, abutment loosening, and fracture of individual attachments on top of a bar. These complications highlight the necessity for regular maintenance, clinician expertise in managing prosthetic complications, and timely intervention to prevent further damage.^33^, ^34^

### Biologic complications

This study aimed to investigate various biologic complications associated with MIS‐ODs and to identify potential risk indicators contributing to their development. The median time to the first biologic complication was 24.5 months. More than half of the MIS‐ODs (55.8%) experienced biologic complications, ranging from peri‐implant mucositis to implant loss. A higher, though not statistically significant, prevalence of peri‐implant mucositis was observed in prostheses utilizing splinted attachment, which aligns with findings reported in prior literature.^4^, ^25^, ^27^ Several factors may account for this association, including poor denture and oral hygiene, ^12^ and limited hygiene access underneath the bar, which can be further compromised when reduced restorative space leads to the bar being positioned abutting the mucosa.^32^ These findings emphasize the importance of prosthetic design considerations that support long‐term peri‐implant tissue health, particularly in patients with reduced restorative space or reduced hygiene compliance.

Implant loss after loading occurred in 14.0% of cases, more frequently with non‐splinted attachments (*n* = 10) than splinted (*n* = 6), a finding contrary to Kern et al., who reported greater loss in splinted designs, though their analysis primarily involved mandibular prostheses.^33^ The difference may be attributed to the higher level of stress around non‐splinted implants.^34^

No significant difference was observed in peri‐implantitis incidence between attachment types, aligning with prior research.^25^, ^35^, ^36^ However, other studies have reported greater marginal bone loss around non‐splinted attachments.^37^, ^38^, ^39^, ^40^ The magnitude of bone loss observed in this study aligns with results reported in several earlier studies.^33^, ^41^ Notably, Payne et al. also found no association between the type of attachment mechanism and marginal bone loss.^42^ The relatively high rate of peri‐implant bone loss (43%) observed may be partly explained by the extended follow‐up period and irregular recall attendance among participants, underscoring the necessity of routine follow‐up.^42^ The method of assessing bone loss—primarily panoramic or periapical radiographs—and the recording of outcomes at the prosthesis rather than individual implant level may have influenced reported prevalence.^43^ These factors should be carefully considered in the interpretation of outcomes and in the design of future longitudinal studies.

### Transition to a new prosthesis

This study found no statistically significant association between patient‐related variables, prosthesis design characteristics, or implant factors and the overall survival of MIS‐ODs, thereby supporting the initial hypothesis that prosthesis and implant survival are not influenced by design characteristics. However, the occurrence of technical and biologic complications, specifically acrylic tooth wear, compromised esthetics, and implant failure after loading the prosthesis demonstrated a strong association with reduced prosthesis survival, leading to rejection of the second hypothesis that survival can be affected by the presence of technical or biologic complications. These results underscore the importance of early identification and management of complications to enhance long‐term success.

Acrylic tooth wear, although not directly detrimental, was significantly associated with prosthesis survival. The relationship may reflect time‐dependent changes rather than mechanical failure. While literature regarding the role of opposing dentition remains inconclusive, ^22^ studies have suggested that natural opposing arches can increase occlusal loads and accelerate tooth wear.^14^, ^30^ Slot et al. reported that over half of IS‐ODs required replacement within 5–10 years, largely due to acrylic wear and discoloration.^44^

The management of acrylic tooth wear depends on severity, ranging from minor maintenance to prosthesis replacement in severe cases. These interventions can significantly influence the average lifespan of MIS‐ODs, as reflected in the present study. The lack of standardized classification systems for occlusal wear represents a limitation in both clinical evaluation and research comparability, highlighting the need for future studies to develop validated classification systems.^14^, ^22^, ^30^

Implant failure following prosthesis loading can substantially affect MIS‐OD survival, with outcomes largely dependent on the position, number, and distribution of the remaining implants. ^13^ A non‐ideal position and distribution or insufficient number of remaining implants may compromise the prosthesis’ stability and retention, ^45^ necessitating further interventions such as addition of palatal coverage or placement of additional implant(s).

Although palatal coverage is often avoided due to patient comfort concerns.^1^ Its inclusion can be beneficial in cases of implant loss, as it enhances retention and load distribution, and partial or strategic palatal coverage may be a viable option in patients at high risk of implant failure.^6^, ^7^ Moreover, palatal coverage can mitigate denture base fractures,^29^, ^30^ decrease functional strain on attachments, and reduce the need for repeated component replacement—thereby improving cost‐effectiveness.^7^, ^27^, ^46^, ^47^

Approximately 17% of implant failures in this study occurred prior to loading, prompting transition from a planned fixed prosthesis to a MIS‐OD as a rescue solution. Such mid‐treatment modifications may overlook critical parameters—such as inter‐arch space, implant angulation, and distribution—that influence future prosthesis prognosis.^3^, ^5^, ^27^ These findings reinforce the necessity of comprehensive diagnostic planning and risk assessment when formulating maxillary prosthetic treatment strategies.

### Limitations and strengths

This study has certain limitations associated with its retrospective cohort design, which analyzed existing dental records to evaluate outcomes such as MIS‐OD failure.^48^ While this approach is appropriate for studying rare outcomes, it is inherently limited by incomplete documentation, delayed or inconsistent data entry, and variability in clinical record‐keeping, all of which can result in missing or incomplete data.^49^ Patients who experienced complications but did not return for care were not captured, possibly underestimating the frequency of complications. Irregular follow‐up, which is common in older populations, and extended observation further limited data consistency. Although the initial calculation estimated a need for 166 prostheses based on an anticipated 15% failure rate, the final dataset included 86 prostheses with an observed failure rate of 27%. The higher‐than‐expected event rate preserved the validity of the multivariable analysis for reliable modeling with three variables despite the smaller overall sample size. This finding is consistent with recommendations that emphasize the number of events, rather than total sample size, as the key determinant of stability in Cox regression, with at least five events per variable generally considered acceptable.^16^, ^17^

Despite these limitations, this study has several strengths, including a long follow‐up period (up to 34.5 years). It is one of the few studies to provide a detailed analysis of treatment characteristics, maintenance, and complications of MIS‐ODs, including survival analysis and potential risk factors. Furthermore, this study addressed limitations in the existing literature by systematically differentiating between maintenance events and complications, and by collecting detailed data on prosthetic, implant, and patient‐related variables, thereby offering a more comprehensive understanding of MIS‐OD outcomes.

Future trials should prospectively evaluate key prognostic factors such as attachment design, palatal coverage, and opposing arch condition, while incorporating cost and maintenance time comparisons with alternative maxillary rehabilitation approaches.

## CONCLUSIONS

Within the study's limitations, MIS‐OD survival was 72.1% and not significantly influenced by prosthesis design. However, the occurrence of technical and biologic complications adversely affected survival. Careful patient selection, meticulous treatment planning, and consistent long‐term maintenance are essential for minimizing complications and optimizing outcomes. Moreover, the effective management of complications is a critical clinical skill that supports the continued functionality of both the prosthesis and its supporting implants.

## CONFLICT OF INTEREST STATEMENT

The authors do not have any financial interest in the companies whose materials are included in this article.
